# Oxytocin Reactivity during a Wilderness Program without Parents in Adolescents

**DOI:** 10.3390/ijerph192315437

**Published:** 2022-11-22

**Authors:** Ayako Morita, Akiko Shikano, Kazuaki Nakamura, Shingo Noi, Takeo Fujiwara

**Affiliations:** 1 Department of Global Health Promotion, Tokyo Medical and Dental University, Tokyo 113-8510, Japan; 2 Research Institute for Health and Sport Science, Nippon Sport Science University, Tokyo 158-8508, Japan; 3 Department of Pharmacology, National Research Institute for Child Health and Development, Tokyo 157-0074, Japan; 4 Department of Social Medicine, National Research Institute for Child Health and Development, Tokyo 157-0074, Japan

**Keywords:** adolescents, child, nature, oxytocin, independence, Japan

## Abstract

While wilderness programs are recognized as a feasible intervention to promote psychological independence in adolescence, little is known about physiological changes. The present study focused on oxytocin, a key hormone for social cognition and behavior, and investigated changes in OT concentrations during a wilderness program among adolescents. Twenty-one 4th–7th graders were separated from parents and immersed with adventures and challenges in the woodlands of Motegi, Tochigi Prefecture, Japan for 31 days, and dataset of 20 boys aged 9–13 years-old were used for analysis. OT concentrations in early morning saliva samples on days 2, 5, 8, 13, 18, 20, 21, 22 and 30 were determined using ELIZA. We performed multi-level regression analyses to compare the OT concentrations before and after solo and team-based survival challenges, and across the nine observational points, adjusting for potential covariates. We found that adolescents increased OT level in a situation where they needed others’ cooperation and support for survival (coefficient: 2.86, SE: 1.34, *p* = 0.033). Further, we found that adolescents gradually decreased their basal OT level during a long separation from parents (coefficient: −0.083, SE: 0.034, *p* = 0.016). A combination of these findings suggest the OT level may be a marker for psychological independence.

## 1. Introduction

Adolescence is marked by profound biological and social transformation [1] and many of the primary psychiatric disorders typically emerge during this time and persist for life [2]. Gaining independence from parents is regarded as the key developmental task in adolescence and a failure to accomplish the task is proposed as an explanation for psychosocial maladjustment [3,4]. Longitudinal studies of adolescent cohorts showed that those who were not encouraged or reinforced to make decisions for themselves and to act on their thought processes and judgements suffer from various problems. In particular, those who were exposed to high levels of psychological control from parents in early adolescence displayed poor self-identity, low peer acceptance, greater behavioral problems and poor mental health [5,6,7], low academic attainment, poor social relationships, as well as poor mental health in later life [7].

One way to nurture independence in adolescents is to provide proper encouragement and reinforcement to explore the world on their own and take responsibility for their decisions in a safe setting [8]. Wilderness programs adopt this approach and immerse participants to experience mental, physical, emotional, and spiritual challenges, such as survival challenges and long-term parental separation, in a safe and supportive outdoor environment [9,10]. A review of the solo wilderness programs reported that participants gain greater sense of independence by being free from manipulation and domination from others [11]. Meta-analyses of the group-based wilderness programs indicated that participation in learning and practicing survival skills and/or high and low adventures, increases self-concept, internal locus of control, self-efficacy and self-reliance and reduces social aggression [9,12]. One study allocated Grade 9th and 10th classes at one boarding school to a full, partial or no wilderness program and found a significant stable improvement in cognitive autonomy relative to the degree of participation [13]. Some studies suggested individual attributes such as age, gender, motivation and past experience may influence the engagement level, reflection and impact of the wilderness program [14,15].

Why the wilderness programs promote independence in adolescents is less understood. Qualitative studies have identified self-reflection and introspection with facilitators playing a vital role in the participants’ psychosocial benefits [16]. On the other hand, the potential physiological mechanisms that support psychological independence remained largely unknown, as there are limited studies which investigated physiological changes during wilderness programs. We hypothesized that unique social contexts of wilderness programs may induce changes in the levels of the hormone oxytocin (OT) which enhances our social cognitions and social behaviors in a manner that urge establishment or maintenance of strong social bonds [17,18,19]. It is widely documented that OT release is increased during exposure to various social stimuli and contexts such as physical and non-physical contacts with another individual [20,21], synchronous group activities [22,23] and unfriendly or hostile environment [24,25]. On the other hand, people who experienced parental divorce in childhood displayed lower OT levels in urine [26] and maternal separation induces long-lasting changes in decreased level of OT receptor binding in the juvenile and adolescent rats’ brain [27]. To our knowledge, however, no previous study has explored the changes in adolescent OT excretions during a wilderness program.

The objective of the study was to investigate changes in oxytocin (OT) concentrations during a wilderness program in adolescents.

## 2. Materials and Methods

### 2.1. Study Participants

We conducted a single armed observational study as part of “Hello Woods”, a 31-day residential wilderness camp organized by Honda Mobilityland Corporation. The required sample size for a mixed model which takes into account of within-individual correlations was calculated to be 19 by the standard formula for paired t-test with a 95% confidence level, a 5% margin of error, a moderate (*r* = 0.4) correlation, and estimated effect size and SD to be 3.0 and 4.0 based on the previous study [28]. In 2015, Hello Woods was offered for around USD2500 (as of 14 March 2022) all inclusive, except for transportation to the camp site for 4th to 7th grade students who are considered as senior students in Japan as our upper primary level education begins from 4th grade. In total, 21 adolescents were recruited online on a first-come-first-served basis during summer school holidays. The vast majority (i.e., 20) were males; therefore, we analyzed the dataset among male participants only.

All the participants and their parents received detailed oral and written communication on the study procedures in person at information sessions prior to the camp, and both provided consent to participate in the study to the program organizer. Research protocol which was prepared in accordance with the principals of the Declaration of Helsinki was reviewed and approved from the institutional review board at the National Center for Child Health and Development (no. 1052).

### 2.2. Program Contents

Hello Woods was taken place in a 42-hectare peri-urban woodlands in Motegi, Tochigi Prefecture, Japan. The program (Table 1) consisted of several basic wilderness survival skill buildings and activities organized in a way to promote understanding about self and others (a team and a group). There were three major survival challenges (A, B, C). The first was “overnight solo bushcraft camping (A)” on days 5–7, whereby participants had to set up a tent for themselves, collect branches and light a fire using matches alone. On days 20–22, participants were divided into three teams and they had to go through “overnight team-based bushcraft camping (B)” They were tasked to set up a tent, collect branches, light a fire using gimlets, cook meals outdoor with a knife and fire, and take turns to keep the fire going all day and night for three days. On days 25–27, the participants experienced “expedition (C)” with the same team members. They had to walk 60 km towards the seaside using a map and compass, while camping and maintaining a fire overnight along the way. All the challenges were performed in the presence of experienced adult camp counselors who ensured nobody would be placed in harm’s way. However, the counselors did not provide direct help to the participants to them to accomplish the tasks. When any participants became upset or frustrated, the counselors would advise them to think why things were not working and to accept unfairness rather than seeking reasons. The counselors also conducted debriefing sessions in the form of recreational activities, such as having the participants write poems and songs, and create drawings based on their experiences during the camp.

### 2.3. Measurements

#### 2.3.1. Oxytocin Level

Saliva samples were collected using the Salivette^®^ cotton roll (Sarstedt, Nümbrecht, Germany) [25,29] at around 5:00 AM on days 2, 5, 8, 13, 18, 20, 21, 22 and 30 (Table 1). To avoid contamination, the participants were instructed to refrain from consuming food and drinks other than water and from brushing teeth for 60 min and 10 min, respectively before sample collection. Collected samples were immediately stored in a freezer at −20 °C and sent to the National Center for Child Health and Development (Tokyo, Japan) at the end of the camp to be kept at −80 °C until analysis. OT concentrations were measured using a commercial ELISA kit (Assay Designs; Ann Arbor, MI, USA) and standardized by total protein content per milliliter and expressed in picograms per milligram protein (pg/mg protein) [28]. Missing information on height and weight (*n* = 1) was replaced with the average of three participants with the same age, and missing information on OT level on day 2 (*n* = 2) and day 18 (*n* = 2) was replaced with subsequently measured OT level of the same participants.

#### 2.3.2. Covariates

Because there are individual differences in OT concentrations and reactivity to laboratory-induced or naturalistic stress due to age, body mass index (BMI), medical use and preexisting medical and psychiatric conditions [21,30,31], a brief questionnaire was administered at the time of application to the participants’ guardians. It included questions about the participants’ age, self-motivation for the camp participation (asked “Who was the one who expressed the wish to participate in the camp?” and coded 1 if the participant’s name was mentioned and 0 if not mentioned), experience of the 31-day residential wilderness program in the past (yes = 1, no = 0), medical use and preexisting medical and psychiatric conditions (“Is your child under any medication or does s/he have any medical and psychiatric illnesses?”), and height and weight.

### 2.4. Analysis

We performed multi-level linear regression model analysis nested within individuals to investigate if salivary OT level changed from just before to during or after the solo and small-group bushcraft camping (day 5 vs. day 9; day 20 vs. day 21 and 22) and over the course of the program (day 2, 5, 8, 13, 18, 20, 21, 22 and 30), using the STATA SE ver. 17 for Windows (STATA, Lightstone Corp., USA). We chose the within-subject design (i.e., multi-level modeling) over the between-subject design (i.e., single-level modeling) because OT levels across the nine time points were likely to be dependent on individuals at a considerable extent, and to increase statistical power [32]. We built models with and without adjusting for potential confounders (i.e., age, sex, the presence of self-motivation for the camp participation, the past experience of residential wilderness program, height and BMI.

## 3. Results

The participants’ characteristics are summarized in Table 2. Their age ranged between 9 and 13 years. None of them were overweight/obese. About half (52.4%) of the participants joined the program with high expectation of its benefits, and five (23.8%) had experiences of the same wilderness program. With respect to OT concentrations in early morning saliva, the highest mean was observed on day 9 (13.6 ± 3.0 pg/md), one day after the end of solo bushcraft survival challenge, and the lowest mean was observed on day 21 (9.5 ± 4.9 pg/md), one day after the team-based bushcraft survival challenge started. The Intraclass Coefficient (ICC) estimate in crude model showed approximately 28.5% of the variance was attributed to between-individual variations, suggesting that the same individuals exhibited similar levels of OT across nine points.

**Figure 1:**
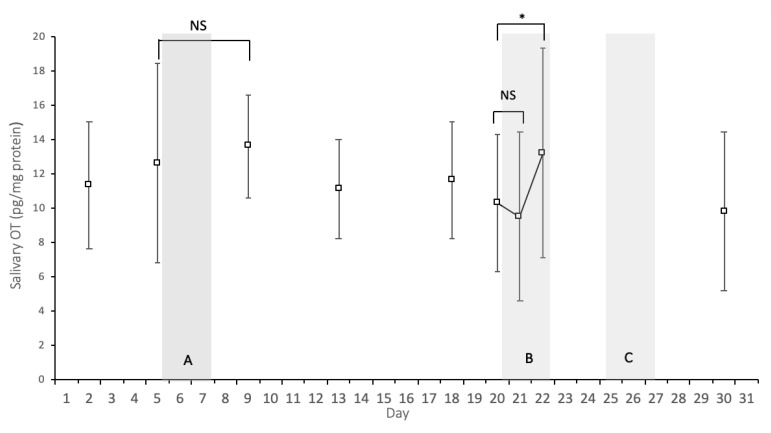
Mean (SD) salivary oxytocin (OT) before and after the solo bushcraft survival challenge (A) and the team-based bushcraft survival challenge (B). Notes: * = *p* < 0.05; NS = *p* ≥ 0.10; (C) is where 60 km expedition was held.

The results of the mixed-effect regression analyses to compare OT levels before and after the bushcraft survival challenges are presented in Figure 1. OT level measured two days after the completion of the solo bushcraft survival challenge was not significantly different from that measured just before the challenge (coefficient = 0.96, standard error (SE) = 1.23, *p* = 0.43). The OT level was also similar on the second day of the team-based bushcraft survival challenge compared just before it stared (coefficient = −0.83, SE = 1.34, *p* = 0.53). However, it was significantly elevated on the third day of the challenge (coefficient = 2.86, SE = 1.34, *p* = 0.033). The coefficient, SE and *p*-value remained the same even after adjusting for the participants’ age, presence of self-motivation at the time of application, history of the wilderness program, height, and BMI.

**Figure 2:**
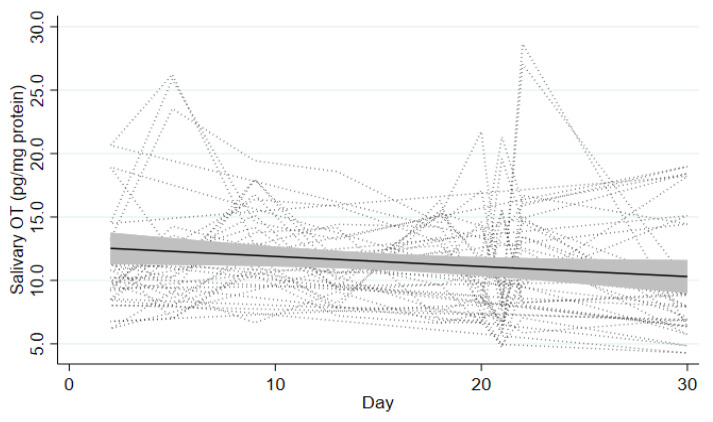
Trend of the oxytocin (OT) release over the course of the 31-day wilderness program Note: The dotted line with gray area indicates a regression line with 95% confidence interval.

Trajectories of OT levels during the wilderness program for the 21 participants is presented in Figure 2. All the participants displayed fluctuation in the OT level for one to two times during the program, with a couple of peaks commonly around the overnight solo bushcraft camping (days 5–7) and group-based bushcraft camping (days 20–22). However, the OT level appears to decline on average towards the end of the program.

Mixed-effect regression analyses showed the statistically significant decline during the whole camping program before and after adjusting for the participants’ age, presence of self-motivation at the time of application, history of the wilderness program, height, and BMI (Table 3). Among the adjusted individual-level factors, height and self-motivated participation was associated with statistically significant declines of OT levels during the camp (coefficient = −0.19, SE = 0.09, *p* = 0.029; coefficient = −2.74, SE = 1.10, *p* = 0.013, respectively).

## 4. Discussion

To the best of our knowledge, the present study is the first to examine changes in OT levels in adolescents during a wilderness program. A total of 20 male adolescents were separated from parents and immersed in high adventures and challenges in the wilderness for 31 days. While we found no significant change in the OT level following the solo bushcraft survival challenge, we found a significant increase in the OT level during the team-based bushcraft survival challenge. We furthermore found a significant gradual decrease in OT level over the course of 31 days.

We observed temporal increase in OT release during team-based bushcraft survival challenges while we did not find a significant increase in OT release following solo bushcraft survival challenge. These finding are consistent with existing reports on OT reactivity in adolescents under various experimental conditions [22,23,25,33]. One of the main functions of OT is to enhance social sensitivity [17,18,19]. OT level has been reported to temporarily increase in response to psychosocial stress in healthy adolescents [25]. At the same time, a number of intervention studies have shown that activities involved interactions with others, such as storytelling, resulted in increased OT release more than activities that did not involve others, such as riddle quizzes for children [22,23,33]. The greater increase in OT level during the team-based survival challenge than the solo survival challenge, therefore, may be due to a combination of psychosocial pressure and presence of others to perform the activity. A study which employed the same technique to analyze salivary OT among Japanese children found a similar level of increase (i.e., 2.63 pg/mg protein) by playful interaction in mothers of first-born boys and the increased maternal OT level to be a significant predictor of lower externalizing problem in children two years later [28].

We also found that OT level in adolescents decreased over time by prospectively measuring OT levels for nine times in total from the same participants. This finding is consistent with the earlier studies that reported lower levels of OT in adults and decreased level of OT receptors in rats experiencing parental separation in childhood [26,27]. A higher OT level is generally regarded to improve social and emotional functioning of the individuals as lower OT level has been reported among people with autism spectrum disorder [34] and victims of severe childhood abuse [35]. However, an increasing number of studies indicates there may be an optimal level of social sensitivity. Experimental studies showed that intranasal OT injection resulted in overrating of emotion in faces in healthy women [36,37], men with low autism spectrum traits [37] and extraverted or prosocial characteristics [38]. In relation to parent–child relationship, a cross-sectional study of 50 healthy mothers found that lower levels of OT was associated with greater provision of autonomy support towards their toddlers [39]. Furthermore, the reduction of the OT over time is consistent with the natural lifespan development of human. A study that compared OT receptor gene expression in brain, considered as a proxy of OT levels, among various aged samples found that the expression peaked in early childhood and then declined from late childhood throughout adolescence and early adulthood, but started to increase again in late-adulthood [40]. Higher OT receptor gene expression in younger children and older adults are hypothesized to reflect social dependence, the need for social support to grow and survive [41]. The estimated reduction of OT level by a month of the wilderness program based on our study would be similar to the level of change by playful interaction in mothers of first-born boys, which were associated with behavioral problems in the children two years later [28]. Although we do not have a directly comparable study that employed the same technique to measure the OT in a similar sample, our study findings may imply that wilderness programs contributed the natural course of OT reductions as part of increasing social independence in a short period of time.

There are several limitations in the present study. First, our participants included early and pre-teens, and our main analyses were limited to boys only due to small sample size of girls. Age and gender may affect how adolescents respond to the wilderness program. For example, boys and girls may have different motivations (e.g., boys may be more likely seeking adventure while girls looking for emotional connections) and different understanding of the program (e.g., boys may be more likely to attribute success to his skills while girls may be more likely to attribute success to luck) [14]. Similarly, older adolescents (aged 12 years and above) may have greater OT reactivity to solitude experiences and separation from parents, although we checked similar point estimate among the older adolescent sample (data not shown due to limited sample size of four). Compared to younger adolescents, they maybe more engaged in reflective practices as they are expected to thrive for emotional independence over personal independence [3] and have better reasoning skills [11]. Furthermore, they likely came from families of high socioeconomic status because the program fee was equivalent to average monthly salary of most Japanese [42]. In order to evaluate the effect of the wilderness program on OT responses, a large study is warranted to investigate whether the OT response changes in girls and a broader group of adolescents. 

Second, our study was a single-arm observational study. A control group (i.e., adolescents who spent summer without participating in the wilderness program) is necessary to affirm that the wilderness program induced the changes in OT excretion level as seasonal change in temperature [43] and the school holiday [44] might have affected their stress levels and OT excretion.

Third, we did not measure psychological independence directly utilizing existing scales such as Psychological Jiritsu Scale [45]. Although independence is set as a common developmental goal throughout the aged 6–18 years-old, personal independence may be emphasized for aged 11 and less and emotional independence from parents may be emphasized for aged 12+ [3,4]. Future studies should measure various types of independence including personal/emotional independence and psychosocial functioning via a questionnaire, and investigate how changes in OT release are associated with changes in different kinds of independence and psychosocial functioning in young (<12) and older (12+) children. 

Fourth, we measured OT in saliva, which encompasses a simple and painless collection method suitable for multiple-time collection from young people [33]. Salivary OT has been criticized for not strongly correlated with plasma oxytocin [46,47]; however, salivary OT testing kits have been substantially improved to reduce non-specific binding to non-OT antibodies and increased salivary OT has been found to associate with function of the social brain regions [48].

Fifth, we measured early morning OT concentrations nine times during the 31 days, but we were not able to measure before or after the program as the participants were gathered from all places in the country. Future studies should follow the participants beyond the program to measure basal and after-the-program OT levels. As a single measurement of salivary OT may not be enough to reflect an individual’s OT system, multiple measurements for an extended period would be recommended [47]. This would allow to evaluate if the observed changes were out of the normal fluctuation of OT levels and long-lasting.

## 5. Summary and Conclusions

In summary, this study is the first to examine the changes in OT concentrations during the wilderness program in adolescent boys. Our findings indicate that adolescents increase OT level in a situation where they need others’ cooperation and support to survive, which is consistent with previous studies that suggested a role of OT in within-group cooperation. Our findings further indicate that adolescents gradually decrease their basal OT level when separated from parents and confronted with adventures and challenges for 31 days. Our exploratory study suggests that OT level may be a marker for psychological independence in healthy adolescents. Further research is warranted to confirm the changes in OT excretion and their association with growth of psychological independence during a wilderness program in a diverse group of adolescents.

## Figures and Tables

**Table 1:** Schedule of the 31-day wilderness program.

1st term: Understanding self		
Day	Main activity	OT sample
1	opening ceremony/Building a basecamp	
2	field walk	#1
3	knife skill building (cutting a fish with a knife)	
4	using a rope to set up a tent and building a fire	
5	overnight solo bushcraft camping (A)	#2
6	overnight solo bushcraft camping (A)	
7	overnight solo bushcraft camping (A)	
8	designing a team flag	
9	skill and team building	#3
10	skill and team building	
2nd term: Understanding others		
Day	Main activity	OT sample
11	canoeing practice and playing in the river	
12	canoeing touring	
13	making a team flag and building a fire	#4
14	motorbiking experience or handicraft making	
15	motorbiking experience or handicraft making	
16	motorbiking experience or handicraft making	
17	motorbiking experience or handicraft making	
18	making a team flag	#5
19	preparing for overnight team-based bushcraft camping	
20	overnight team-based bushcraft camping (B)	#6
21	overnight team-based bushcraft camping (B)	#7
22	overnight team-based bushcraft camping (B)	#8
23	cleaning up after (B)	
24	preparing for walking expedition	
25	60 km expedition (C)	
26	60 km expedition (C)	
27	60 km expedition (C)	
28	cleaning up after walking expedition	
29	knife maintenance	
30	farewell party	#9
31	closing ceremony	

**Table 2:** Characteristics of the male participants (*n* = 20).

Variable		*n* (%)	Mean ± SD	Range	
Age (years)			10.6 ± 1.2	9	13
	9 years	4 (20.0)			
	10 years	6 (20.0)			
	11 years	6 (30.0)			
	12 years	2 (30.0)			
	13 years	2 (10.0)			
Height (cm)			134.0 ± 8.4	125	163
BMI			16.9 ± 2.1	13.4	21.1
Presence of self-motivation for the camp					
	No	10 (50.0)			
	Yes	10 (50.0)			
History of the wilderness program participation					
	No	15 (75.0)			
	Yes	5 (25.0)			
The early morning salivary OT (pg/mg)					
	#1 (day 2)		11.3 ± 3.7	6.2	20.7
	#2 (day 5) *		12.6 ± 5.8	7.0	26.2
	#3 (day 9)		13.6 ± 3.0	6.7	19.4
	#4 (day 13)		11.1 ± 2.9	7.2	18.6
	#5 (day 18)		11.6 ± 3.4	6.6	16.3
	#6 (day 20) **		10.3 ± 4.0	6.6	21.7
	#7 (day 21) **		9.5 ± 4.9	4.7	21.3
	#8 (day 22) **		13.2 ± 6.1	4.9	28.7
	#9 (day 30)		9.8 ± 4.6	4.3	19.0

Note: * = day when overnight solo bushcraft camping (A) was conducted, ** = a day when overnight team-based bushcraft camping was conducted (B).

**Table 3:** Adjusted mixed effects of multi-level regression model for salivary oxytocin level (pg/mg) during the one-month summer camp (*n* of bushcraft survival challenges [level 1] = 2, *n* of observations [level 2] = 180, *n* of the individuals [level 3] = 20).

Variables	Crude Model		Adjusted Model	
	Coefficient (SE)	*p*	Coefficient (SE)	*p*
Time-level				
Day	−0.084 (0.035)	0.015	−0.083 (0.034)	0.016
Individual-level				
Age (year)	−0.49 (0.41)	0.23	−0.16 (0.57)	0.78
Height (cm)	−0.14 (0.057)	0.015	−0.19 (0.09)	0.007
BMI	−0.081 (0.24)	0.73	0.065 (0.24)	0.78
Self-motivation (ref = no)	−2.09 (0.92)	0.022	−2.81 (0.093)	0.003
History (ref = no)	−1.05 (1.13)	0.35	0.84 (1.15)	0.46

Note: Crude estimate of cons and residual is estimated based on the model where day and presence of survival challenge were entered as exposure.

## Data Availability

Data is available upon reasonable request and approval from the National Center for Child Health and Development.

## References

[1] Sawyer S.M., Azzopardi P.S., Wickremarathne D., Patton G.C. (2018). The age of adolescence. Lancet Child Adolesc. Health.

[2] Solmi M., Radua J., Olivola M., Croce E., Soardo L., Salazar de Pablo G., Il Shin J., Kirkbride J.B., Jones P., Kim J.H. (2022). Age at onset of mental disorders worldwide: Large-scale meta-analysis of 192 epidemiological studies. Mol. Psychiatry.

[3] Havinghurst R. (1972). Developmental Tasks and Education.

[4] Hochberg Z., Belsky J. (2013). Evo-devo of human adolescence: Beyond disease models of early puberty. BMC Med..

[5] Pettit G.S., Laird R., Dodge K.A., Bates J.E., Criss M.M. (2001). Antecedents and behavior-problem outcomes of parental monitoring and psychological control in early adolescence. Child Dev..

[6] Boudreault-Bouchard A., Dion J., Hains J., Vandermeerschen J., Laberge L., Perron M. (2013). Impact of parental emotional support and coercive control on adolescents’ self-esteem and psychological distress: Results of a four-year longitudinal study. J. Adolesc..

[7] Loeb E.L., Kansky J., Tan J.S., Costello M.A., Allen J.P. (2021). Perceived Psychological Control in Early Adolescence Predicts Lower Levels of Adaptation into Mid-Adulthood. Child Dev..

[8] Sanders R.A. (2013). Adolescent Psychosocial, Social, and Cognitive Development. Pediatr. Rev..

[9] Hattie J., Marsh H.W., Neill J.T., Richards G.E. (1997). Adventure education and outward bound: Out-of-class experiences that make a lasting difference. Rev. Educ. Res..

[10] Jong M., Lown E.A., Schats W., Mills M.L., Otto H.R., Gabrielsen L.E., Jong M.C. (2021). A scoping review to map the concept, content, and outcome of wilderness programs for childhood cancer survivors. PLoS ONE.

[11] Naor L., Mayseless O. (2020). The Wilderness Solo Experience: A Unique Practice of Silence and Solitude for Personal Growth. Front. Psychol..

[12] Hans T.A. (2000). A Meta-Analysis of the Effects of Adventure Programming on Locus of Control. J. Contemp. Psychother..

[13] Margalit D., Ben-Ari A. (2014). The Effect of Wilderness Therapy on Adolescents’ Cognitive Autonomy and Self-efficacy: Results of a Non-randomized Trial. Child Yuth Care Forum.

[14] McKenzie M. (2000). How are adventure education program outcomes achieved? A review of the literature. Aust. J. Outdoor Educ..

[15] Hogrel J.-Y., Decostre V., Alberti C., Canal A., Ollivier G., Josserand E., Taouil I., Simon D. (2012). Stature is an essential predictor of muscle strength in children. BMC Musculoskelet. Disord..

[16] Purc-Stephenson R.J., Rawleigh M., Kemp H., Asfeldt M. (2019). We Are Wilderness Explorers: A Review of Outdoor Education in Canada. J. Exp. Educ..

[17] Quintana D.S., Lischke A., Grace S., Scheele D., Ma Y., Becker B. (2021). Advances in the field of intranasal oxytocin research: Lessons learned and future directions for clinical research. Mol. Psychiatry.

[18] Ebert A., Brüne M. (2018). Oxytocin and Social Cognition. Curr. Top. Behav. Neurosci..

[19] Takayanagi Y., Onaka T. (2021). Roles of Oxytocin in Stress Responses, Allostasis and Resilience. Int. J. Mol. Sci..

[20] Filippa M., Monaci M.G., Spagnuolo C., Serravalle P., Daniele R., Grandjean D. (2021). Maternal speech decreases pain scores and increases oxytocin levels in preterm infants during painful procedures. Sci. Rep..

[21] Uvnäsmoberg K., Ekström-Bergström A., Buckley S., Massarotti C., Pajalic Z., Luegmair K., Kotlowska A., Lengler L., Olza I., Grylka-Baeschlin S. (2020). Maternal plasma levels of oxytocin during breastfeeding—A systematic review. PLoS ONE.

[22] Yuhi T., Kyuta H., Mori H.A., Murakami C., Furuhara K., Okuno M., Takahashi M., Fuji D., Higashida H. (2017). Salivary Oxytocin Concentration Changes during a Group Drumming Intervention for Maltreated School Children. Brain Sci..

[23] Rassovsky Y., Harwood A., Zagoory-Sharon O., Feldman R. (2019). Martial arts increase oxytocin production. Sci. Rep..

[24] Fujiwara T., Kubzansky L.D., Matsumoto K., Kawachi I. (2012). The Association between Oxytocin and Social Capital. PLoS ONE.

[25] Bernhard A., van der Merwe C., Ackermann K., Martinelli A., Neumann I.D., Freitag C.M. (2018). Adolescent oxytocin response to stress and its behavioral and endocrine correlates. Horm. Behav..

[26] Boccia M.L., Cook C., Marson L., Pedersen C. (2021). Parental divorce in childhood is related to lower urinary oxytocin concentrations in adulthood. J. Comp. Psychol..

[27] Lukas M., Bredewold R., Neumann I., Veenema A. (2010). Maternal separation interferes with developmental changes in brain vasopressin and oxytocin receptor binding in male rats. Neuropharmacology.

[28] Nawa N., Nakamura K., Fujiwara T. (2020). Oxytocin Response Following Playful Mother–Child Interaction in Survivors of the Great East Japan Earthquake. Front. Psychiatry.

[29] Bernhard A., Kirchner M., Martinelli A., Ackermann K., Kohls G., Gonzalez-Madruga K., Wells A., Fernández-Rivas A., De Artaza-Lavesa M.G., Raschle N.M. (2021). Sex-specific associations of basal steroid hormones and neuropeptides with Conduct Disorder and neuroendocrine mediation of environmental risk. Eur. Neuropsychopharmacol..

[30] Weingarten M.F.J., Scholz M., Wohland T., Horn K., Stumvoll M., Kovacs P., Tönjes A. (2019). Circulating Oxytocin Is Genetically Determined and Associated with Obesity and Impaired Glucose Tolerance. J. Clin. Endocrinol. Metab..

[31] Tabak B.A., Leng G., Szeto A., Parker K.J., Verbalis J.G., Ziegler T.E., Lee M.R., Neumann I.D., Mendez A.J. (2022). Advances in human oxytocin measurement: Challenges and proposed solutions. Mol. Psychiatry.

[32] van IJzendoorn M.H., Bakermans-Kranenburg M.J. (2016). The Role of Oxytocin in Parenting and as Augmentative Pharmacotherapy: Critical Issues and Bold Conjectures. J. Neuroendocrinol..

[33] Brockington G., Moreira A.P.G., Buso M.S., da Silva S.G., Altszyler E., Fischer R., Moll J. (2021). Storytelling increases oxytocin and positive emotions and decreases cortisol and pain in hospitalized children. Proc. Natl. Acad. Sci. USA.

[34] Huang Y., Huang X., Ebstein R.P., Yu R. (2021). Intranasal oxytocin in the treatment of autism spectrum disorders: A multilevel meta-analysis. Neurosci. Biobehav. Rev..

[35] Goh K.K., Lu M.-L., Jou S. (2021). Childhood Trauma and Aggression in Persons Convicted for Homicide: An Exploratory Study Examines the Role of Plasma Oxytocin. Front. Psychiatry.

[36] Cardoso C., Ellenbogen M.A., Linnen A.-M. (2014). The effect of intranasal oxytocin on perceiving and understanding emotion on the Mayer-Salovey-Caruso Emotional Intelligence Test (MSCEIT). Emotion.

[37] Bartz J.A., Nitschke J., Krol S.A., Tellier P.-P. (2019). Oxytocin Selectively Improves Empathic Accuracy: A Replication in Men and Novel Insights in Women. Biol. Psychiatry Cogn. Neurosci. Neuroimaging.

[38] Human L., Thorson K., Mendes W. (2016). Interactive effects between extraversion and oxytocin administration: Implications for positive social processes. Soc. Psychol. Pers. Sci..

[39] Miura A., Fujiwara T., Osawa M., Anme T. (2014). Inverse Correlation of Parental Oxytocin Levels with Autonomy Support in Toddlers. J. Child Fam. Stud..

[40] Rokicki J., Kaufmann T., de Lange A.-M.G., van der Meer D., Bahrami S., Sartorius A.M., Haukvik U.K., Steen N.E., Schwarz E., Stein D.J. (2022). Oxytocin receptor expression patterns in the human brain across development. Neuropsychopharmacology.

[41] Audunsdottir K., Quintana D. (2022). Oxytocin’s dynamic role across the lifespan. Aging Brain.

[42] (2020). The Japanese Ministry of Health Labour and Welfare, Summary Report on Basic Survey on Wage Structure (Nationwide). www.mhlw.go.jp/toukei/itiran/roudou/chingin/kouzou/z2020/dl/13.pdf.

[43] Barreca A., Schaller J. (2020). The impact of high ambient temperatures on delivery timing and gestational lengths. Nat. Clim. Chang..

[44] Gao M., Havitz M.E., Potwarka L.R. (2018). Exploring the Influence of Family Holiday Travel on the Subjective Well-being of Chinese Adolescents. J. China Tour. Res..

[45] Takasaka Y., Toda H. (2006). Psychological Jiritsu in Adolescence (II): Construction of Psychological Jiritsu Scale. J. Hokkaido Univ. Educ..

[46] Quintana D.S., Westlye L.T., Smerud K.T., Mahmoud R.A., Andreassen O.A., Djupesland P.G. (2018). Saliva oxytocin measures do not reflect peripheral plasma concentrations after intranasal oxytocin administration in men. Horm. Behav..

[47] Martins D., Gabay A.S., Mehta M., Paloyelis Y. (2020). Salivary and plasmatic oxytocin are not reliable trait markers of the physiology of the oxytocin system in humans. eLife.

[48] Abraham E., Gilam G., Kanat-Maymon Y., Jacob Y., Zagoory-Sharon O., Hendler T., Feldman R. (2017). The Human Coparental Bond Implicates Distinct Corticostriatal Pathways: Longitudinal Impact on Family Formation and Child Well-Being. Neuropsychopharmacology.

